# Effectiveness of Smartphone App for the Treatment of Pediatric Obesity: A Randomized Controlled Trial

**DOI:** 10.3390/children11101178

**Published:** 2024-09-27

**Authors:** Giuseppina Rosaria Umano, Mariapia Masino, Grazia Cirillo, Giulia Rondinelli, Francesca Massa, Giuseppe Salvatore R. C. Mangoni di Santo Stefano, Anna Di Sessa, Pierluigi Marzuillo, Emanuele Miraglia del Giudice, Pietro Buono

**Affiliations:** 1 Department of the Woman, the Child, and General and Specialized Surgery, University of Campania Luigi Vanvitelli, 80138 Naples, Italy; giuseppinarosaria.umano@unicampania.it (G.R.U.); mariapia.masino@studenti.unicampania.it (M.M.); grazia.cirillo@unicampania.it (G.C.); giulia.rondinelli@unicampania.it (G.R.); massafrancesca96@gmail.com (F.M.); giuseppesalvatorer.c.mangonidis.stefano@unicampania.it (G.S.R.C.M.d.S.S.); anna.disessa@unicampania.it (A.D.S.); pierluigi.marzuillo@unicampania.it (P.M.); 2 Maternal and Child Health Department, Directorate-General for Health, 80143 Naples, Italy; pietro.buono2@regione.campania.it

**Keywords:** obesity, treatment, m-Health, children and adolescents, mobile app

## Abstract

Background: Pediatric obesity treatment is based on high-intensity lifestyle counseling. However, high dropout rates and low effectiveness have been reported, even in specialized centers. Mobile health technologies have been used to overcome these limits with contrasting results. This study aims at evaluating the effectiveness of a six-month intervention with a mobile app for the treatment of pediatric obesity at 6 and 12 months of follow-up. Methods: Seventy-five patients were randomly assigned to standard care or standard care plus mobile app (2:1) using an online randomizer system. The mobile app delivered high-intensity lifestyle counseling for diet and physical activity. Results: At six months of follow-up, the M-App group showed significantly lower dropout rates compared to standard care (*p* = 0.01). The risk of dropout was significantly higher in controls compared to the intervention group (OR 3.86, 95% C.I. 1.39–10.42, *p* = 0.01). After one year, we observed lower albeit non-statistically significant dropout rates in the M-App compared to the standard care group (*p* = 0.24). No differences were observed in z-score BMI and percentage of BMI reduction between the two groups. Conclusions: Our findings suggest that the mobile app might help in the clinical management of children and adolescents with obesity in terms of dropout reduction.

## 1. Introduction

Obesity in children and adolescents is a major public health concern globally. The increasing prevalence of obesity in children has been observed in both developed and developing countries [1]. The prevalence of pediatric obesity has increased dramatically over the past five decades. According to recent estimates, for children aged 5–19 years, the number rose from 31 million in 1990 to 160 million in 2022 [1].

Similar trends are observed worldwide, with the steepest increases occurring in low- and middle-income countries with a slightly higher prevalence in boys [1].

This trend poses a severe threat to future public health since children and adolescents with obesity are more likely to develop obesity-related comorbidities (namely cardiovascular disease, type 2 diabetes, non-alcoholic fatty liver disease, depression, and others) [2,3,4,5,6,7,8,9,10].

Obesity treatment first aims to prevent the development of chronic diseases and reduce adiposity, improving physical and psychosocial comorbidities. Therapy integrates multiple components including nutrition, exercise, psychological counseling, pharmacotherapy, and surgical procedures [11]. The first approach for pediatric obesity consists of lifestyle modifications, including diet, physical activity, and eating behaviors [12].

Scientific societies [12,13] suggest that changes in dietary habits (avoiding the consumption of calorie-dense, nutrient-poor foods, encouraging the consumption of whole fruits) and physical activity education (20 min, optimally 60 min, of vigorous physical activity at least 5 days per week) should be placed at multilevel treatments involving children and adolescents, parents, and communities to empower treatment effectiveness [14]. Other therapeutic strategies are anti-obesity medication and metabolic/bariatric surgery [12]. The current guidelines [14] highlight the relevance of counseling intensity to achieve lifestyle modifications in children and adolescents with obesity. They suggest at least 26 “contact” hours of counseling over 3–12 months to guarantee this high-intensity approach [14]. However, this is not commonly applicable in clinical settings for health professionals and families. Moreover, even if lifestyle treatment is the first approach, it is not always successful. Several studies have reported high dropout rates in several lifestyle interventions for children and adolescents with obesity [15,16,17]. The major barriers to treatment compliance include low socioeconomic status, distance from the clinic, psychological comorbidities, and parents with obesity [15,16,17]. These data highlight the importance of personalizing the intervention according to the family and patient needs and of developing new strategies for treatment delivery. In this light, electronic health (e-Health) resources are a promising tool.

In a clinical trial, Hagman et al. [18] demonstrated that a digital support system with a personalized weight-loss target curve and daily weight measurements shared by the family and the clinic was more effective in terms of weight loss and dropout rates than the standard care for the treatment of children and adolescent with obesity aged 4 to 17 years.

Digital tools can help children with obesity improve their health through different strategies [19]. Moreover, mobile health systems (m-Health) can monitor physical activity, dietary intake, and sleep patterns, thus providing real-time feedback [19].

Some digital tools can facilitate remote coaching and telehealth consultations, allowing healthcare providers to offer personalized advice and support without the need for frequent in-person visits [18].

Several studies have demonstrated improvements in self-reported diet, physical activity, and short-term weight loss [20,21,22,23,24]. However, scientific evidence for the effectiveness of these devices as an adjunct tool for the treatment of pediatric obesity is poor. To date, few studies have been conducted on children and adolescents with obesity. A study by Browne and colleagues reported the low effectiveness of a mobile app in the reduction in attrition in children and adolescents aged 9–16 years [25]. Conversely, as reported above, Hagman reported the superiority of the mobile app in weight loss and attrition after one year of follow-up [18]. Moreover, the available trials are highly heterogeneous in terms of type of intervention (education, behavioral therapy, self-monitoring, and others), sample size, and study population.

Based on this knowledge, this prospective study aims to investigate the effectiveness of a mobile app for smartphones (M-App) for the lifestyle treatment of children with obesity compared to standard care. In detail, the main objectives of the study were as follows: (1) verify the differences in dropout rates between control and intervention groups; (2) assess the differences in weight loss between M-App and standard care groups at 6 and 12 months of follow-up; and (3) assess the changes in diet and physical activity.

## 2. Materials and Methods

### 2.1. Study Design and Participants

The study is a prospective monocentric two-arm open-label randomized controlled trial. The intervention group received standard care provided by a trained pediatrician and a dietitian plus a smartphone app for the treatment of pediatric obesity. The control group received only standard care. Children and adolescents aged 6–12 years attending the pediatric obesity outpatient clinic of the University Hospital “Luigi Vanvitelli” were prospectively enrolled. According to international guidelines [14], obesity was defined based on body mass index (BMI), calculated as the ratio between the weight expressed in kilograms and the height squared expressed in meters (kg/m^2^). Patients were eligible if (1) they had a BMI ≥ 95th percentile for age and sex according to the WHO growth charts [26,27]; (2) they were not taking medications affecting body weight; (3) their parents owned and were able to use a smartphone; and (4) they gave the consent to participate. Children affected by secondary forms of obesity (i.e., endocrinopathies, syndromic obesity, monogenic forms) were excluded. Written informed consent from parents and children to participate was collected before the randomization procedure. The study was conducted according to the Declaration of Helsinki. The Ethical Institutional Review Board of the University of Campania “Luigi Vanvitelli” approved the study (protocol n. 14258/2023). All participants underwent a complete clinical examination as reported elsewhere [28]. Briefly, the child was examined wearing undergarments. Height was measured using a Harpenden stadiometer to the nearest 0.1 cm. Weight was assessed using a balance beam scale to the nearest 0.1 kg. Z-score BMI was calculated with the lambda-mu-sigma method [29]. Waist circumference was measured at the midpoint between the lowest rib and the iliac crest with an anelastic tape, and the average of two values was obtained. The ratio between waist and height (WHR) in centimeters was calculated as an indirect measure of truncal adiposity. Blood pressure was measured three times, and the average of the three measurements was reported. Elevated blood pressure was defined for systolic and/or diastolic blood pressure ≥ 90th percentile for age and sex according to the recent guidelines [30]. After baseline assessment, participants were randomly allocated to standard care or intervention with a 2:1 ratio using an online randomizer system (http://www.graphpad.com/quickcalcs/index.cfm, accessed on 31 May 2023). Randomization was centralized and provided by a different investigator that was not involved in patient enrollment. Sequentially numbered envelopes were used to implement the random allocation. The complete auxological and clinical examinations were performed at 6 months and 12 months of follow-up according to international recommendations [14]. All the clinical examinations were performed using the same balance beam scale and Harpenden stadiometer at the University Hospital “Luigi Vanvitelli”.

### 2.2. Study Intervention

The control group received standard lifestyle care, consisting of nutritional and behavioral counseling provided by dieticians and pediatricians. During the first visit, patients and their families received a treatment plan based on a personalized physical activity plan (according to patient age, ability, and fitness level) and a balanced Mediterranean diet. Moreover, education about junk food, sugar-sweetened beverages, and processed food was delivered to the families and increased consumption of fibers, fruits, and vegetables was encouraged. In addition, psychological counseling for the patient and the family was provided to identify potential comorbidities and provide behavioral therapy.

Follow-up visits were scheduled every three months according to standard care and outpatient clinic waiting lists and included clinical, nutritional, physical activity, and psychological assessment.

The intervention group received the standard care plus the smartphone app (Nutrilio) [Nutrilio FREE, Version 1.17.4, 2023] for six months. After the allocation to the intervention group, parents were invited to download the app through the AppStore (iOS) or Google Play (Android) and received information on how to use the app; children and adolescents did not download the app on their smartphones.

Nutrilio is a free mobile health app designed to help subjects monitor diet and physical activity to improve their overall health. Moreover, it allows individuals to set personal goals to promote a healthy lifestyle. The app is designed with an intuitive interface. It is composed of a section where users record dietary choices (type and amount of food, emotional eating, and also add photos). The main functionality of the app is food tracking, but in addition, Nutrilio offers features to track water intake, exercise, and mental wellbeing. The app provides daily, weekly, and monthly reports to monitor progression over time. Nutrilio also includes customizable reminders to help users stay on track with their health goals.

In our study, parents were asked to complete a daily diary of diet and physical activity together with the child for both diet and exercise and to export it as .csv file. Moreover, they were invited to send this file once a week to the medical staff. After receiving the diary, the medical staff gave feedback to the family about the lifestyle behavior and potential improvements to be applied at home. According to this design, the M-App enabled an educational family-based intervention with high-intensity counseling focusing strictly on lifestyle changes. This approach differs from other scientific evidence for m-Health intervention in pediatric settings that includes an auto-monitoring strategy [18], cognitive-behavioral approach [25], and different age ranges (above 12 years) [31].

In-person visits were scheduled every three months as for the control group. The follow-up was completed after 12 months (6 months with smartphone app and 6 months with standard care alone).

### 2.3. Study Outcomes

The primary outcome of the study was to assess the difference in dropout rates at 6 and 12 months of follow-up between the two groups. Dropout was defined as the presence of less than 1 visit in 6 months. Secondary outcomes were changes in body weight including (1) changes in z-score BMI. Delta changes in z-score BMI were calculated at 6 and 12 months of follow-up compared to baseline evaluation, and differences in absolute z-score BMI pre and post-intervention were also calculated for each group; (2) a clinically significant reduction in baseline BMI defined as a reduction of at least 5% of BMI after 6 and 12 months [32,33]; a clinically significant reduction in z-score BMI was defined as a reduction of at least 0.25 units of z-score BMI at 6 and 12 months compared to baseline [34], and a clinically significant reduction in body weight was defined as a reduction of at least 5% of body weight after 6 and 12 months [35]; (3) a reduction in waist-to-height ratio as surrogate of abdominal adiposity. Moreover, lifestyle changes, including dietary habits and increased levels of physical activity were recorded during the follow-up visits at 6 and 12 months.

### 2.4. Statistical Analysis

Continuous variables were checked for normality using the Kolmogorov–Smirnov test. Differences between groups for continuous variables were investigated by the Whitney U test. Differences for continuous variables for matched pairs were assessed by the Wilcoxon Signed Rank test. Chi-square or Fisher exact tests were used to test differences for categorical variables as appropriate. Data are expressed as a median (interquartile range, IQR) or percentages. Univariate logistic regression analysis was performed to calculate the odds ratio (OR) for dropout according to the allocation group. Moreover, a multivariate logistic regression analysis including age, gender, BMI, and z-score BMI was performed to correct the effect of the allocation group for potential confounding factors. A *p*-value < 0.05 was considered statistically significant. All the analyses have been performed using SAS^®^ on Demand for Academics (SAS Institute Inc., Cary, NC, USA).

The study power was calculated based on a post hoc analysis considering the main outcome of differences in dropout rates between the two groups using G*Power. On the basis of the differences in dropout rates at 6 months between standard care (72%) and M-App intervention (40%), with a sample size of 75 participants, our study has a power of 78% to detect a difference in dropout between groups with an alpha level of 0.05 (calculations have been performed using G*Power Version 3.1.9.6) [36].

## 3. Results

**Figure 1:**
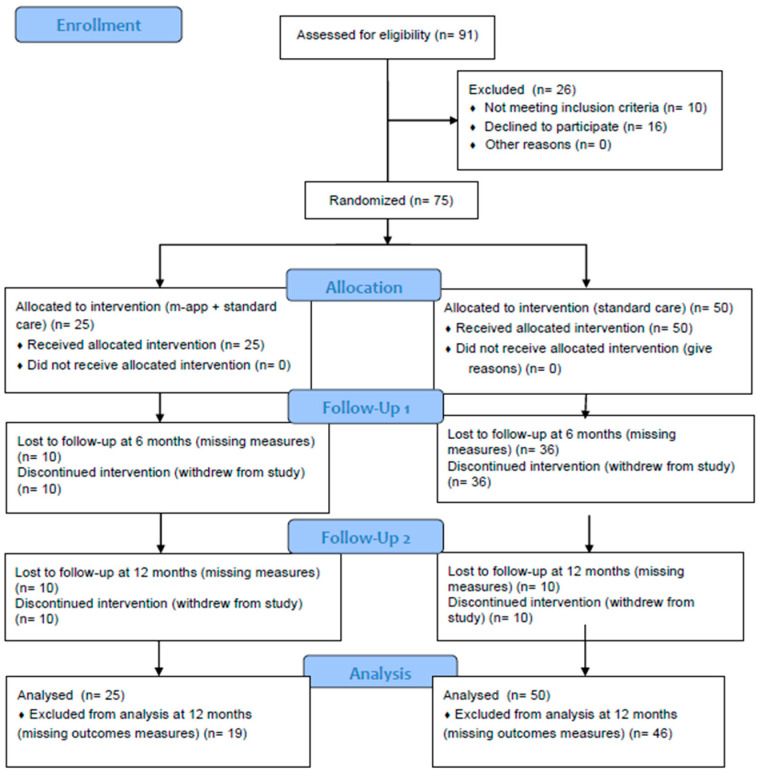
CONSORT flow diagram adapted for the study.

Seventy-five children and adolescents were enrolled (thirty-six males, mean age 9.4 ± 1.5 years). The participants’ enrollment and flow are summarized in the CONSORT diagram (Figure 1) and the characteristics of the entire cohort are displayed in Table 1.

Fifty patients were allocated in the control group and twenty-five were allocated in the intervention group. The two groups did not differ in age, sex, BMI, and BMI z-score distribution (Table 2). In the M-App group, 15 participants (60%) showed good compliance with the study and sent the Nutrilio export data to the medical staff.

### 3.1. Dropout Assessment

**Figure 2:**
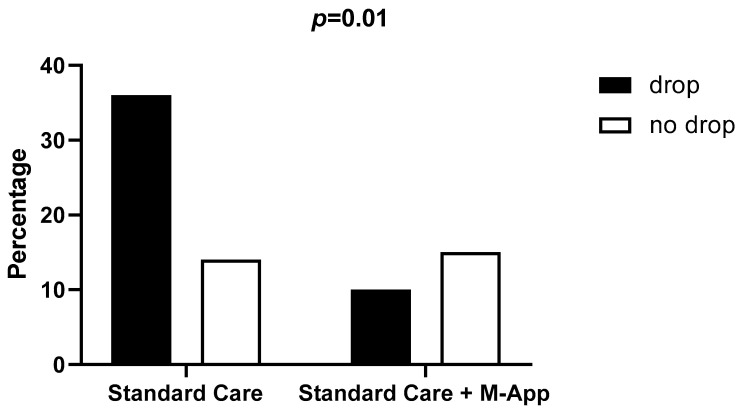
Dropout and 6-month follow-up rates according to allocation group.

After six months, 14 patients in the control group (28%) and 15 patients in the intervention group (60%) completed the follow-up. The dropout rate was significantly higher in the control group (72%) compared to the intervention group (40%, *p* = 0.02) (Figure 2).

Children who completed the follow-up at 6 months did not significantly differ at baseline for age, gender, BMI, z-score BMI, and blood pressure compared to those who did not show up at follow-up (Table 3).

The univariate logistic regression analysis revealed that the standard care group showed a 3.86-fold higher risk for dropout compared to the M-App group (OR 3.86, 95% C.I. 1.39–10.42, *p* = 0.01). This risk was independent of the effect of age, gender, BMI, and z-score BMI (*p* = 0.008). At one year of follow-up, there were no differences in the dropout rate between the two groups (*p* = 0.24); the standard care showed a 92% and the intervention group showed an 80% dropout rate.

### 3.2. Weight-Loss Outcomes

**Figure 3:**
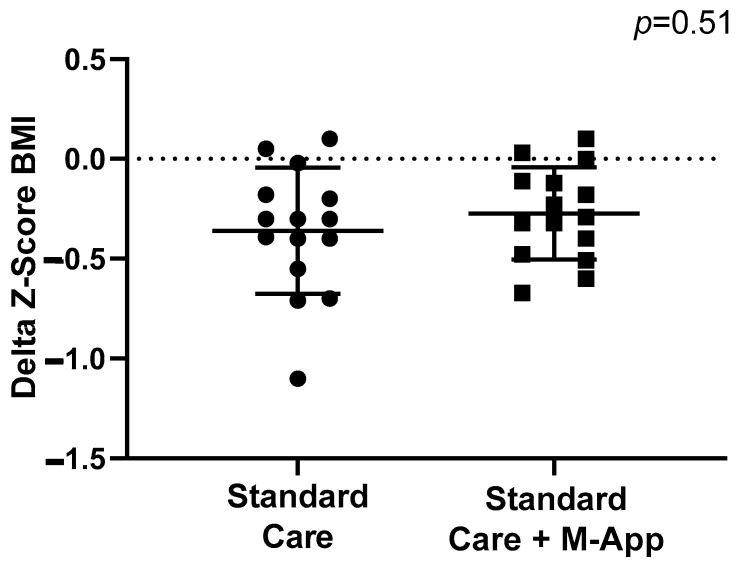
Differences in delta z-score BMI at 6-month follow-up between standard care and M-App groups.

Weight-loss achievement was evaluated as a reduction in z-score BMI, a reduction in baseline BMI in percentage, a reduction in body weight, and as a reduction in WHR. The two groups showed similar z-score BMI reduction at 6 months of follow-up (*p* = 0.51). The median reduction in z-score BMI in the standard care group was −0.3 units (IQR −0.55; −0.18) and −0.29 (IQR −0.48; −0.11; see Figure 3) in the M-App group. Moreover, the rates of a clinically significant reduction (−0.25 units) in z-score BMI did not differ between the two groups (standard care 66.7% vs. M-App 53.3%; *p* = 0.71). No differences were observed at 12 months in delta z-score BMI reduction between the two groups (*p* = 0.99) and absolute BMI (median reduction 0.64 in standard care vs. 0.60 in the M-App group, *p* = 0.91). However, the M-App group showed a significantly lower z-score BMI (*p* = 0.0006) at 6 months (2.8, IQR 2.5–3.0) compared to baseline (3.0, IQR 2.8–3.4). This difference was not statistically significant in controls (3.3 IQR 3.2–3.9 at baseline vs. 3.3, IQR 2.6–3.4 at 6 months; *p* = 0.13). The z-score BMI was stable at the 12-month compared to 6-month visit in the M-App (*p* = 0.25) and standard care groups (*p* = 0.50).

**Figure 4:**
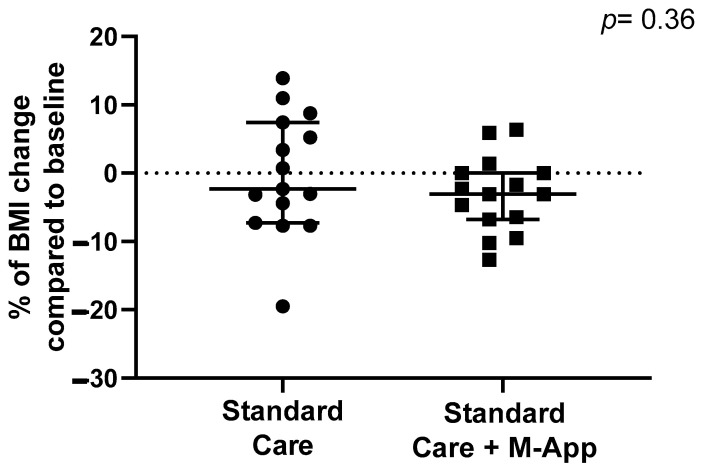
Differences in BMI percentage reduction at 6-month follow-up between standard care and M-App groups.

The median reduction in BMI percentage after 6 months was 3.75% (IQR 0.0%; 7.63%). There were no differences in BMI percentage change between the two groups (*p* = 0.36). The median change in BMI in the standard care group was −2.3% (IQR −7.3%; 7.1%) vs. −3.1% (IQR −6.8%; 0.0%; see Figure 4) in the M-App group. The rate of patients achieving at least 5% of BMI reduction compared to baseline did not differ between the two groups (53.3% of controls vs. 33.3% of cases; *p* = 0.46). Moreover, the two groups showed a similar BMI percentage change at 12 months of follow-up (*p* = 0.90). The rate of patients achieving at least 5% of body weight reduction compared to baseline was similar in the two groups (21.4% in controls and 26.7% in cases, *p* = 0.75) at 6 months and 12 months of follow-up (one patient in each group, *p* = 0.89). Similarly, no differences were observed in WHR changes between the two groups at 6 months [standard care: −0.02 (IQR −0.04, 0.00); intervention: −0.02 (IQR −0.05, −0.00); *p* = 0.58] and 12 months [standard care: 0.02 (IQR −0.06, −0.06); intervention: 0.00 (IQR −0.06, 0.03); *p* = 0.81]. None of the patients showed a reduction in the WHR below 0.50. The rate of elevated blood pressure was evaluated; after 6 months of follow-up, the intervention group showed a lower prevalence of hypertension compared to the control group (30% vs. 60%); however, this difference was not statistically significant (*p* = 0.36). Elevated blood pressure regressed in 10% of controls and 30% of cases (*p* = 0.58). Gender dimorphisms were assessed for anthropometric measures at baseline, dropout at 6 and 12 months, and weight-loss measures. However, no differences were observed between males and females.

### 3.3. Lifestyle Changes Assessment

Both groups reported a similar trend in any lifestyle change at 6 months (53.3% of intervention and 46.7% of standard care group, *p* > 0.99). However, the M-App group more frequently reported dietary changes—increased intake of fruits and vegetables and a lower intake of sugar-sweetened beverages (53.3%)—compared to the standard care (13.3%, *p* = 0.05), whereas the standard care group more frequently reported an increase in physical activity (33.3%) compared to the intervention group (20.0%, *p* = 0.68). After 1 year of follow-up, an overall rate of 66.7% for any lifestyle change was reported, with a greater albeit not significantly higher prevalence in the M-App group (80%) compared to the standard care group (50%, *p* = 0.58). No adverse events were reported in both groups.

## 4. Discussion

In this study, we investigated the effectiveness of a smartphone app (Nutrilio) in improving lifestyle intervention compliance in children and adolescents with obesity. The intervention lasted 6 months followed by 6 months of follow-up without the use of the smartphone app. We observed a significantly lower dropout rate in the M-App group compared to the standard care after the first six months of follow-up. However, this finding was not confirmed at 12 months of follow-up. The regression analysis confirmed that the use of the mobile app was protective against the risk of dropping out during follow-up independently of other confounding factors included in the analysis.

Attrition represents a common concern in the treatment of obesity, both in childhood and adulthood [37,38]. Elevated attrition rates and poor compliance to treatment reduce the effectiveness of clinical interventions for lifestyle modifications, and subsequently for weight-loss achievement. Moreover, dropout is associated with poor disease control and negative health outcomes in the medium and long term. Therefore, to improve the success of pediatric weight management programs, new strategies to counteract the dropout phenomenon should be promoted. Several studies have investigated the predictors of attrition during lifestyle intervention trials with heterogeneous results [17,39,40]. The rates of dropout are generally high and tend to vary according to the type of intervention (diet, physical activity, diet plus physical activity). In common clinical practice, the dropout rate in pediatric obesity is around 60% after one year of treatment [38,41]. Among predictors, low socioeconomic status, parental obesity, lower z-score BMI at baseline, distance from the hospital, adolescence, and male gender have been associated with a higher risk of dropout [17,39]. Conversely, the presence of comorbidities, such as hyperglycemia, insulin resistance, and dyslipidemia have been associated with higher compliance to treatment. This is probably due to more consciousness about obesity as a disease from both parents and patients [39].

In this context, m-Health can be a strategy to increase adherence. This theory is supported by the study by Roth et al. [42], who described a dropout rate of 19.7% after 12 months of intervention, which is lower than the dropout rate reported in other studies (ranging from 26.8 to 39%) in 150 adults with obesity. However, it is difficult to estimate the realistic effects of m-Health because of the high rate of dropouts. In fact, a high rate of attrition was found in different studies about m-Health interventions reducing the positive outcomes of intervention in children and adolescents aged 9–16 years [25,43]. The study by Desmet et al. [31] focused on the attrition rate during an m-Health intervention in adolescents with obesity aged 12–16 years, which also included a psychological assessment. In this study, dropouts were associated with demographic factors (age, gender, and socioeconomic status), health-related factors (baseline weight, initial motivation levels, and prior experience with technology), and the feasibility of the app (features and support). No statistically significant difference in attrition was found across the different eating styles. The only significant negative predictor of attrition was adjusted BMI: a higher adjusted BMI may result in greater motivation to use m-Health apps [31]. In the study of Hagman et al., children and adolescents aged 4–18 years with the M-App showed similar attrition rates compared to standard care patients. Among predictors, the older age range (above 12 years) was associated with higher attrition [18]. In our study, we included younger children (age range 6–12 years) with major involvement of parents in the intervention and M-App use groups. Therefore, our study differs from the previous studies in population and intervention type. We did not find predictors of dropout except for M-App use. This finding might be related to the small sample size and lack of information about the socioeconomic status that has underpowered the ability to find other predictors.

Moreover, we evaluated the effect of the mobile app on weight-loss outcomes. Weight loss was assessed as both z-score BMI and BMI percentage reduction over time at 6 and 12 months. The two groups displayed a similar delta changes in z-score BMI, body weight, and BMI values over time. However, only the M-App group showed a significantly lower z-score at 6 months compared to baseline. Therefore, in our study, the M-App intervention was partially superior to standard care in inducing a more clinically significant weight loss. These findings might suggest that the M-App is useful in intensifying counseling and reducing the attrition rate, but not in increasing the subject’s adherence to lifestyle modifications. The scientific literature has reported that a 0.25 to 0.5 unit reduction in z-score BMI is associated with an improvement of cardiometabolic risk in children and adolescents with obesity [34]. In a cohort of about 1400 children with obesity, Reinehr et al. reported that a reduction of at least 0.125 units of z-score BMI was associated with an improvement of several cardiovascular risk factors (hypertension, dyslipidemia, insulin resistance). This effect was more pronounced for a z-score reduction of 0.25 units after 1 year of lifestyle intervention in the Obeldicks trial [33]. Previous studies conducted in smaller groups have reported that a z-score BMI lowering of 0.5 units might be beneficial for patients in terms of the resolution of comorbidities [44]. Conversely, another study reported a clinical benefit for a 0.01 unit decrease in systolic and diastolic blood pressure levels [45]. In our study, we did not perform blood tests to assess insulin levels, dyslipidemia, and glucose homeostasis. However, we observed a trend for reduction in elevated blood pressure rates that was more pronounced in the M-App group compared to the standard care groups. Nevertheless, this finding was not statistically significant. Therefore, we could not draw any conclusion about the cardiometabolic risk reduction or the best z-score decrease cut-off for cardiometabolic profile improvement in our cohort.

In addition, during the last few years, the measures of clinically meaningful weight loss have been widely discussed. In clinical practice, current guidelines recommend using BMI for obesity diagnosis as a surrogate of body adiposity and the z-score BMI represents an estimate of obesity severity [12,13,14]. However, when the z-score BMI is measured in cohorts with many participants above the 97th percentile, the clinical utility for change over time is limited. The z-score values for BMI above the 97th percentile are based on calculations and therefore might be less accurate [32]. Moreover, for severe obesity, the z-score values are compressed with a narrow spectrum of variability even for significant changes in BMI values. This phenomenon reduces the impact of z-score reduction after treatment, leading to an underestimate of the treatment efficacy over time [32]. Therefore, other metrics for treatment outcomes estimates have been proposed, including the percentage change in body weight [35], the absolute and percentage change BMI, the change in percent of the median, the reduction of 5–10–15% in BMI, and others [33]. However, the application of these other metrics is scarce in pediatric research, limiting the possibility of comparing study outcomes and different intervention efficacies. In our study, we included the metric of percentage change in BMI and the reduction of at least 5% in baseline BMI and weight after 6 months. As for the z-score BMI decrease, we found no differences in terms of weight-loss assessment between the two groups.

In our study, the positive effect of the M-App on treatment compliance was lost after M-App use discontinuation. This is in line with previous studies that reported no weight-loss maintenance and/or weight regain after intervention discontinuation in children aged 6–11 years [46,47]. Few studies have been published on the long-term effectiveness of behavioral interventions for pediatric obesity [46,48]. The scientific literature suggests that a longer duration (at least 6 months) and younger ages (below 6 years of age) are associated with better results that could be maintained over time [48,49]. A recent study reported a persistence of weight loss in the participants of the More or Less Trial that included younger children aged 4 to 6 years and their parents after 48 months of follow-up [48].

We acknowledge the presence of limitations that constrict the generalizability of our findings. The lack of a validated protocol for the measurement of anthropometric measurements might limit the reproducibility of the results. Moreover, the relatively short duration of the M-App intervention (less than 1 year) might have limited the ability to detect the long-term effect of the treatment. In addition, the small sample size might have underpowered the study in identifying predictors of dropout in our cohort. In particular, the lack of information about parental socioeconomic status reduces the ability to identify all the major predictors of dropout. Moreover, the subjects included in the study were homogeneous in terms of age, gender, and BMI distribution; therefore, we did have not enough power to detect other risk factors for attrition to lifestyle intervention in pediatric obesity. However, at the same time, this also represents a strength of the study. In fact, we were able to assess the independent effect of the mobile application as an add-on therapy in the management of children and adolescents with obesity without other confounding factors.

To date, the available scientific evidence is highly heterogeneous, and for clinicians, it is hard to draw clear conclusions about the effectiveness of e-health technologies in clinical practice, especially in pediatric age.

## 5. Conclusions

This study showed that a mobile app with an educational component and feedback activity was effective in reducing the attrition rate during a lifestyle intervention for pediatric obesity. This effect was limited to the duration of the use of the mobile app and was not confirmed after M-App use interruption. No effects were observed on weight-loss achievement compared to standard care.

These findings suggest that m-Health technologies represent a promising tool as a support in clinical practice to empower lifestyle counseling intensity. Moreover, these tools allow healthcare professionals to perform a multilevel approach targeting both parents and children, as advocated by current guidelines. However, the dropout rate over time continues to be high and the strength of these findings should be further confirmed in larger studies.

## Figures and Tables

**Table 1:** Characteristics of the entire cohort.

Parameter	Data
Subjects (n)	75
Male (%)	48
Age (IQR) [years]	9.40 ± 1.50
BMI (IQR) [kg/m^2^]	27.60 ± 4.10
Z-score BMI	3.10 ± 0.69
Systolic Blood Pressure [mmHg]	113 ± 15
Diastolic Blood Pressure [mmHg]	69 ± 9
Waist Circumference [cm]	85 ± 10
Waist-to-Height ratio	0.59 ± 0.06

**Table 2:** Characteristics of the groups according to intervention.

	Standard Care + M-App	Standard Care	*p*
Subjects (n)	25	50	
Male (%)	44	50	0.81
Age (IQR) [years]	10.60 (8.30–10.90)	9.30 (8.00–10.30)	0.20
BMI (IQR) [kg/m^2^]	28.10 (25.20–29.50)	26.80 (24.30–29.90)	0.99
Z-score BMI (IQR)	2.9 (2.8–3.3)	3.3 (2.55–3.65)	0.32
Systolic Blood Pressure (IQR) [mmHg]	111 (105–118)	111 (100–125)	0.45
Diastolic Blood Pressure (IQR) [mmHg]	67 (59–75)	70 (65–80)	0.14
Elevated blood pressure (%)	46	36	0.68
Waist Circumference (IQR) [cm]	86 (79–90)	84 (78–91)	0.97
Waist-to-Height ratio (IQR)	0.58 (0.56–0.6)	0.61 (0.55–0.65)	0.14

**Table 3:** Baseline characteristics according to dropout and 6-months of follow-up.

	Dropped-Out	Returned at Follow-Up	*p*
Subjects (n)	46	29	
Male (%)	58	41	0.48
Age (IQR) [years]	9.6 (8.4–10.7)	9.1 (8.1–10.6)	0.35
BMI (IQR) [kg/m^2^]	26.9 (24.8–31.1)	28.3 (24.0–29.7)	0.40
Z-score BMI (IQR)	3.0 (2.4–3.5)	3.2 (2.9–3.7)	0.27
Systolic Blood Pressure (IQR) [mmHg]	113 (104–125)	111 (100–125)	0.72
Diastolic Blood Pressure (IQR) [mmHg]	71 (60–75)	70 (60–80)	0.85
Waist Circumference (IQR) [cm]	84 (79–89)	89 (77–94)	0.27
Waist-to-Height ratio (IQR)	0.60 (0.57–0.63)	0.59 (0.54–0.65)	0.47

## Data Availability

Data are available upon request to the corresponding author.
